# Pathological Section of Medico-Legal Society

**Published:** 1894-02

**Authors:** Clark Bell, Edward C. O’Brien

**Affiliations:** Vice-Chairman and Secretary; Treasurer


### Annual Report of the Psychological Section of the Medico-Legal Society.

*To the Fellows of the Medico-Legal Society:*

The summary of the work of this Section for the year 1893, is briefly stated as follows:

There have been fifty-three members elected. The bulletin of the Section has been published of which four numbers of Vol. I have been issued, which are herewith submitted as a part of the report.

These contain a record of the scientific work of the Section.

By-laws, rules and regulations for the government of the Section and management of its affairs were adopted on November 11, 1893, and approved by the Executive Committee of the Medico-Legal Society, November 14, 1893.

The following change has been made in the organization of the officers of the Section, viz.: Nine vice-chairmen (additional) have been elected, instead of one vice-chairman, to take their position as such January 1894.

The following are the present officers of the Section:

Chairman—Prof. Elliot Coues, of Washington, D. C.

Vice-Chairmen—Clark Bell, of New York; A. E. Osborne, M. D., of California; Harold Browett, of China; James T. Searcy, M. D., Alabama; F. E. Daniel, M. D., of Texas; T. D. Crothers, M. D., Hartford, Conn.; Henry Hulst, M. D., of Michigan; R. J. Nunn, M. D., of Georgia; U. O. D. Wingate, M. D., of Ala.

Secretary—Clark Bell, Esq.

Treasurer—Edward C. O'Brien, Esq.

Executive Committee—Clark Bell, Chairman; Prof. Elliott Coues, Edward C. O'Brien.

All members of the Medico-Legal Society are eligible to membership in this Section at annual subscription of $1.50 each.

All others, who are approved by the Executive Committee, on payment of $5 initiation fee and $1.50 annual dues, payable in advance, and all members of the Section receive the Bulletin free. The wives of members of the Medico-Legal Society are eligible to membership on payment of $1.50 annual dues, with-initiation fee.

Respectfully submitted on behalf of the Section,

CLARK BELL,
Vice-Chairman and Secretary.

EDWARD C. O'BRIEN, Treasurer.

